# Lifestyle Interventions in Overweight and Obese Pregnant or Postpartum Women for Postpartum Weight Management: A Systematic Review of the Literature

**DOI:** 10.3390/nu10111704

**Published:** 2018-11-07

**Authors:** Kathryn V. Dalrymple, Angela C. Flynn, Sophie A. Relph, Majella O’Keeffe, Lucilla Poston

**Affiliations:** 1 Department of Women and Children’s Health, School of Life Course Sciences, King’s College London, 10th Floor North Wing, St Thomas’ Hospital, Westminster Bridge Road, London SE1 7EH, UK; Angela.flynn@kcl.ac.uk (A.C.F.); Sophie.relph@kcl.ac.uk (S.A.R.); lucilla.poston@kcl.ac.uk (L.P.); 2 Department of Nutritional Sciences, School of Life Course Sciences, King’s College London, Franklin-Wilkins Building, 150 Stamford Street, London SE1 9NH, UK; Majella.okeeffe@kcl.ac.uk

**Keywords:** lifestyle interventions, maternal obesity, postpartum weight retention

## Abstract

Excessive gestational weight gain (GWG) and postpartum weight retention (PPWR) may predispose women to the development of obesity. The objective of this systematic review was to evaluate the effectiveness of lifestyle interventions in overweight or obese pregnant and/or postpartum women for managing postpartum weight up to 2 years after giving birth. Eighteen randomised controlled trials were included (2559 participants) and divided into three categories according to the timing of the intervention: pregnancy only (*n* = 3), postpartum only (*n* = 12) and pregnancy and postpartum (*n* = 3). The intervention duration varied from 10 weeks to 10 months and included diet only (*n* = 5) or diet and physical activity (*n* = 13). Seven postpartum only interventions reported significant improvements in postpartum weight when compared to the control group. Most of these interventions were short and intensive, lasting 10–16 weeks. One pregnancy only and one pregnancy and postpartum intervention reported reduced PPWR at 6 months. Nine trials did not report an effect of the intervention on postpartum weight. However, of these, four reported associations between GWG and PPWR. This review suggests that successful postpartum weight management is achievable with intensive lifestyle interventions starting in the postpartum period; however, there is insufficient evidence to conclude whether interventions starting in pregnancy are effective. Larger trials utilising comparative methodologies in the pregnancy and postpartum periods are required to inform the development of targeted strategies preventing PPWR or reducing postpartum weight.

## 1. Introduction

Obesity is a global epidemic. In the UK, over 60% of adults are categorised as overweight or obese [1] and cumulative prevalence is predicted to reach 70% by 2034 [2]. Of Particular concern, is the fact that 49.6% of pregnant women in England are categorised as overweight or obese [3]. It is established that obesity reduces fertility and in pregnancy increases the risk of adverse maternal, fetal and neonatal outcomes, but it is less well appreciated that pregnancy per se is a major contributor to the development of obesity, through the retention of weight gained during pregnancy [4,5].

Estimates of postpartum weight retention (PPWR) vary, with as many as 20% of women reported to retain more than 5 kg after one year postpartum [6,7]. Excessive gestational weight gain (GWG), above that recommended by the Institute of Medicine (IOM) guidelines, has consistently been reported as a predictor of PPWR [8,9,10,11] and a contributor to the obesity epidemic among women [12,13,14,15]. Furthermore, pre-pregnancy body mass index (BMI) also contributes to PPWR; women who enter pregnancy overweight or obese are at greater risk of exceeding the IOM GWG [16,17,18] and are less likely to return to their pre-pregnancy weight compared to their normal weight counterparts [19]. One study reported that between the first two pregnancies, an increase of BMI ≥4 kg/m^2^ occurred in 7.5% of normal weight women, 10.5% of overweight women, and 13.4% of obese women [20]. Importantly, PPWR has been associated with an increased risk of many adverse outcomes in a subsequent pregnancy independent of the woman’s initial BMI [21,22,23].

In the UK, the current NICE guidelines for weight management after pregnancy recommend breastfeeding as a strategy for promoting weight loss [24], but findings from studies of women with heterogenous BMI are mixed, as reported in a systematic review [25] in which it was suggested that pre-pregnancy BMI and GWG may have greater influence on postpartum weight.

Given the increase in prevalence of overweight and obesity among women of reproductive age, placing them at an increased risk of PPWR, it is important to investigate the impact of interventions in this group during the pregnancy and/or postpartum period in this at-risk group. The antenatal and postnatal periods are well established windows of opportunity for public health interventions, due to the increased contact with healthcare professionals [26]. Earlier systematic reviews of women of heterogenous BMI showed that lifestyle interventions based on diet and/or physical activity are effective at either preventing excessive GWG [27,28] or reducing PPWR [12,29,30]. This study was undertaken to update the previous reviews and to consider the timing of the intervention in women who are overweight or obese, which has not previously been addressed. Furthermore, there is inadequate data to support the implementation of any specific approach which can be translated and implemented into clinical practice. Our aim was to systematically evaluate the effectiveness of lifestyle interventions initiated during the antenatal and/or postnatal period in overweight or obese pregnant women to manage postpartum weight.

## 2. Methods

This review paper has been reported in accordance with the PRISMA guidelines for reporting a systematic review [31] and registered on 5 June 2018 on the PROSPERO database for systematic reviews (PROSPERO 2018: CRD42018096480).

### 2.1. Literature Search

A literature search was conducted by two independent reviewers using three databases: MEDLINE, Embase and the Cochrane Central Register of Controlled Trials. The search strategy, listed in Appendix A, of keywords and Medical Subject Headings (MeSH) terms was adapted to each database. All randomised controlled trials published in English between 1 January 2000–23 January 2018 were included. The timeframe was selected to reflect up to date knowledge for overweight and obesity in the antenatal and postnatal period. A hand search of reference lists in identified articles and review articles was performed to identify additional relevant studies.

### 2.2. Inclusion and Exclusion Criteria

The inclusion and exclusion criteria were developed using the PICOS [32] (population, intervention, comparison, outcomes and study design) approach, summarised in Table 1. Inclusion criteria included: (1) randomised controlled study design, (2) diet or diet and physical activity intervention initiated anytime during pregnancy or up to 2 years postpartum, including trials which aimed to manage gestational diabetes mellitus (GDM), (3) the inclusion of participants with a BMI > 25 kg/m^2^ and, if applicable, these being analysed separately to women with a healthy BMI, (4) outcome data collected after a minimum of 3 months postpartum to ensure GWG did not influence follow-up weight, and (5) changes in the mother’s body composition up to 2 years after giving birth (e.g., change in weight from baseline, reduction in postpartum weight retention or changes to BMI). Studies were excluded if they were non-randomised trials, observational studies and cohort analyses of maternal outcomes, or if they included women with underlying disorders diagnosed pre-pregnancy, such as type 1 diabetes or hypertension, women with multiple pregnancies, interventions in women with a BMI < 25 kg/m^2^, or if women younger than 18 years of age were included.

### 2.3. Study Selection and Data Extraction

Following the removal of duplicates, titles and abstracts were independently screened against the inclusion/exclusion criteria by two researchers. Data extraction was completed separately but systematically by two reviewers and included general characteristics (title, authors, date and place of publication), recruitment strategy, number of participants, intervention details (type, duration and frequency) and relevant outcome measures, including primary and secondary outcomes relating to body composition and weight and breastfeeding outcomes. Data extraction from the retrieved papers was carried out between March 2018 and August 2018.

### 2.4. Data Synthesis

Data was analysed using quantitative and qualitative techniques, through a narrative summary approach to aid the interpretation of trial results. A meta-analysis was precluded due to the heterogeneity of the studies with regards to study design, intervention methodologies and outcome measures [33]. Studies were partitioned into three groups depending on the timing of the intervention (1) pregnancy only, (2) pregnancy and postpartum and (3) postpartum only. This grouping enabled the exploration of the association between intervention initiation and postpartum weight reduction.

### 2.5. Assessment of Validity and Bias

The Cochrane Handbook for Systematic Reviews of Interventions tool [34] was used to assess the validity and bias of each included publication. The domains used included randomisation selection (selection bias), allocation concealment (selection bias) and participant drop-out between recruitment and follow-up period (attrition bias). Performance bias was not included due to the nature of the interventions. The studies were scored in each of these domains as having a ‘high’, ‘low’, or ‘unclear’ risk of bias and an overall risk was subsequently determined following the Cochrane guidelines.

## 3. Results

**Figure 1:**
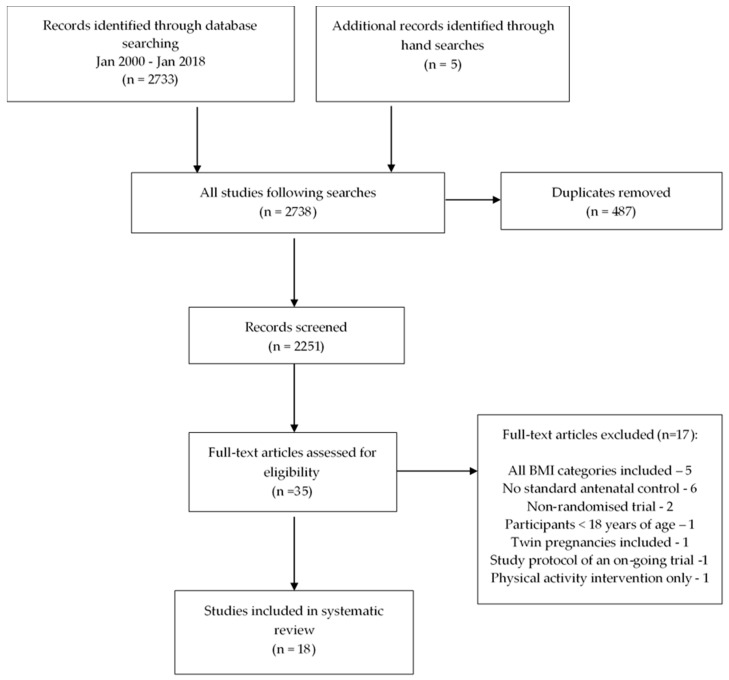
Flowchart of study selection.

A total of 2733 titles were identified in the initial database search (Medline and Embase 2456, Cochrane 277) and five additional titles were identified from hand searches (Figure 1). Following the removal of duplicates, 2251 titles and abstracts were screened and the full texts of 35 studies were assessed for eligibility; 18 met the inclusion criteria. Five studies were excluded as they failed to provide a separate overweight (OW) or obese (OB) subgroup analysis; six studies had no standard antenatal control arm; a further two studies were excluded as they were non-randomised trials, one trial included twin pregnancies, one included participants under 18 years of age, one intervention was of physical activity only and one publication was a protocol for an ongoing trial.

The characteristics of the trials are summarised in Table 2. The eighteen studies (*n* = 2559) are divided into three categories according to the timing of the intervention: pregnancy only (*n* = 3) [35,36,37], pregnancy and postpartum (*n* = 3) [38,39,40] or postpartum only (*n* = 12) [41,42,43,44,45,46,47,48,49,50,51,52].

The primary aim of each study was variable. The majority of the studies aimed to reduce postpartum weight (*n* = 14) [35,36,37,38,39,40,41,43,45,46,47,49,50,51], three studies aimed to improve diet quality [42,44,52] and one trial aimed to determine whether weight loss affected infant growth [48]. In those studies where the assessment of postpartum weight management was not included as a primary aim, it was reported as a secondary outcome [42,44,48,52]. All trials initiated during pregnancy (*n* = 6) reported changes in weight from pre-pregnancy weight to 6 [35,37,39,40] and/or 12 months postpartum [36,38,39]. One of these trials set individual weight reduction goals of 5% lower than pre-pregnancy weight [38]. The postpartum-only trials (*n* = 12) reported on changes in weight from baseline to the end of the intervention [41,42,43,44,45,46,47,48,50,51,52] or a subsequent follow-up [49], with two trials also reporting on changes in weight at follow-up from reported pre-pregnancy weight [49,51]. 

With regards to the study populations, the sample size ranged from 18 [46] to 450 participants [50]. Recruitment ranged from the first trimester [37] of pregnancy to 18 months postpartum [43]. Two trials focused on women with GDM [38,49], two focused on socially disadvantaged or low-income women [43,46], one on African–American women [39] and three recruited lactating women only [41,42,48].

Intervention content included a combined approach of diet and physical activity (*n* = 13) [35,36,37,38,39,41,42,43,45,46,48,49,50] or diet only (*n* = 5) [40,44,47,51,52]. The type of contact during the interventions ranged from face-to-face (*n* = 4) [36,37,40,51], technology-based (*n* = 4) [39,45,46,49], both face-to-face and technology-based (*n* = 8) [35,38,41,42,43,44,47,50] or postal delivery of educational kits (*n* = 1) [52]. One trial did not detail the type of contact during the intervention [48]. The intensity of the face-to-face interventions varied from one session at the start intervention [35,47,51] to at least three contact times per week [42] throughout the intervention period. The interventions using technology-only and educational kits also varied from daily [45,46] to monthly contact [52]. The intervention duration varied from 10 weeks [48] to 10 months [52] both of these trials were from the postpartum period only.

The studies used a variety of strategies to modify dietary intake. The approaches included dietary counselling [35,36,37,44,45,51], the American Diabetes Association (ADA) diet [38], diet-focused behaviour change goals (e.g., to limit junk food intake) [39], a low-carbohydrate diet [40], an energy-restrictive diet [41,43,46,47,48], a low-GI/high-fibre diet [49], decrease in calorie-dense foods and increase in fruit and vegetables [50], access to the MyPyramid website [42] and educational kits which focused on dietary habits, including portion sizes, fruit and vegetable intake, nutritious snacks and reading food labels [52]. Of the 13 trials which included a physical activity component, strategies to modify activity included advice to exercise daily for 30 min per day or a step count [36,45], exercise classes [37,42,48,50], weekly exercise goals of 150 min per week [38,43,49] and daily walking [35,39,41,46]. Four trials provided the participants with weight loss goals, which were either weekly [48], end of intervention [38,47,49] or personalised to each participant [45,46].

The standard care group advice was diverse: eight studies reported that the control group received ‘standard care’ [35,37,38,39,40,41,44,46]. Five studies reported that the control group received ‘standard care’ with the additional specification of routine interventions for overweight or obese women, including one session with a dietitian [36], advice and services as part of the Women, Infant and Children (WIC) program [45], written information on weight loss [43] or the provision of healthy eating brochures [47,49] and information on nutrition for breastfeeding [51]. For the remaining four studies, the control groups were given instructions not to restrict energy intake or perform structured exercise throughout the intervention [42,48] or received bi-weekly [50] and monthly newsletters on reading skills and enjoyment for the preschooler [52].

The earliest assessment of postpartum weight occurred at 14 weeks postpartum [48] and the latest assessment was performed at 2 years following birth [43]. Breastfeeding outcomes were also reported in 12 studies [35,37,38,41,42,44,45,46,48,49,51,52]. Interventions were delivered by individuals with varying qualifications, ranging from dietitian [37,38,40,41,42,45,47,49,51], lifestyle counsellor [43,50], health coach [39,46,52], nutrition professional [44] or an interventionist [35,36,45]. One trial did not provide details on who delivered the intervention [48].

### 3.1. Effect of Interventions on Postpartum Weight Retention

Of the 18 trials, nine reported a significant effect on PPWR weight when compared to controls (Table 3). The largest reduction in postpartum weight of 6.1 kg (−8.4 to −3.2 kg) followed a 12-week postpartum diet-only intervention [47]. Of these nine effective trials, one focused on a diet-only approach [47] and eight focused on diet and physical activity [41,42,43,45,46,48,49,50]. The nine trials include seven postpartum interventions (total OW/OB participants *n* = 390) [41,42,43,46,47,48,49], one intervention initiated during pregnancy (total OW/OB participants *n* = 200) [35] and one which commenced in pregnancy and continued to 6 months postpartum (total OW/OB participants *n* = 66) [39]. The one effective pregnancy only intervention did not show a difference between the intervention and control groups for GWG at the end of pregnancy in OW/OB women, but did demonstrate an increase in the percentage of women who returned to their pre-pregnancy weight: I = 25.6%, C = 16.7% at 6 months postpartum [35]. In the only study which showed an effect from a combined pregnancy and postpartum intervention, there was significant improvement in the percentage of women who were at their pre-pregnancy weight at the end of the intervention (6 months postpartum) I = 56% vs. C = 29%, however this was not maintained by the 12-months follow-up [39].The remaining nine trials did not show an effect of the intervention on changes in postpartum weight when compared to control groups [36,37,38,40,44,45,50,51,52].

For the effective interventions, dietary strategies incorporated reducing calorie intake by up to 500 kcal/day [41,42,43,46,47,48], individualised weight loss goals [46], returning to pre-pregnancy weight [49], weekly weight loss of 0.5–1.0 kg/week [48], end of intervention weight loss goals of −6.0 kg [47] and healthy eating with nutritional counselling [35]. Successful interventions included technology-based strategies in three trials [39,46,49] or both face-to-face and technology-based approaches in eight trials [35,41,42,43,47]. One trial did not detail the type of contact during the intervention [48]. 

Four trials (pregnancy only *n* = 2 [36,37]; pregnancy and postpartum *n* = 2 [38,39]) reported on significant associations between GWG and PPWR; two studies reported a positive association between GWG and PPWR [36,39]. Two studies reported an association between appropriate GWG within in the IOM guidelines [53] and lower PPWR [37,38]. Two studies reported that breastfeeding influenced postpartum weight retention [35,37]. Vinter et al. [37] reported that exclusive breastfeeding significantly negatively influenced PPWR (regression coefficient: −2.64, *p* = 0.002) and Phelan et al. [35] reported that breastfeeding was related to higher odds of achieving 6-month preconception weight or below (OR: 2.4; 95% CI: 1.4, 4.2; *p* = 0.002).

### 3.2. Study Quality

The overall quality of the included studies varied and is summarised in Table 4. Nine trials were classified as having a low risk of bias [35,38,39,41,42,46,47,48,49]. Seven trials were categorised as having a moderate risk of bias [36,37,40,43,45,50,51] and two trials as having a high risk [44,52]. The main source of bias across all the studies was participant attrition, with eight trials reporting at least a 30% drop-out from recruitment to the reported outcomes [36,37,40,43,44,50,51,52].

## 4. Discussion

The aim of this study was to systematically summarise the effectiveness of lifestyle interventions, initiated during the antenatal and/or postnatal period, in OW/OB pregnant women up to 2 years after birth. The findings suggest that interventions which commenced during the postpartum period were effective in reducing postpartum weight; however, due to the small number of trials, we are unable to draw a conclusion about the effect of interventions which start during pregnancy. Therefore, we are unable to comment on the best intervention timing for the management of postpartum weight. Larger trials, with similar and comparable methodologies within the antenatal and postnatal periods, are required to inform the development of targeted strategies aimed at preventing PPWR and/or reducing postpartum weight.

Previous studies have shown that weight retention between pregnancies is associated with the development of obesity [54,55] and the postpartum period has been positioned as an opportune time to engage women in promoting healthy weight and lifestyle changes [56]. This observation is particularly important in overweight and obese women, who are more likely to remain obese one year after giving birth, compared to women of normal weight [57]. Furthermore, an increase in weight and BMI between pregnancies is associated with significantly higher maternal and fetal/neonatal complications in subsequent pregnancies [58].

In this systematic review, seven of the 12 studies from the postpartum period were successful at reducing postpartum weight. However, there was significant heterogeneity in the timing of the initiation of the interventions, which ranged from 2 weeks [46] to 18 months [43] postpartum. It is clear from these results that there is no consensus on the optimal time to engage women in postpartum weight management, but interventions initiated during the postpartum period are effective. Another commonality of successful interventions was intervention intensity; successful studies generally included dietary and physical activity components and target goals with frequent contact either text message or phone call [41,42,43,47] and, in most cases, face-to-face interaction [41,42,43,47] and or interval contact via web application [42,45,49]. This is in line with a previous systematic review of lifestyle interventions of postpartum women with heterogenous BMI [13].

Within this systematic review, the trial reporting the greatest average loss of postpartum weight was a diet-only intervention which included a calorie reduction of 500 kcal/day, and a weekly and end-of-trial weight loss goal of 0.5 kg and 6 kg, respectively. A diet-only approach is reflective of successful weight loss interventions in general [59] and weight management interventions in pregnancy [60]. Of the successful trials, six were of a short duration (10–16 weeks) during the postpartum period [41,42,43,46,47,48] and one was a web-based intervention of 6 months [49]. Whilst the short intervention approach was successful in the short term, only three of these trials reported weight loss maintenance at follow-up visits of 12 months postpartum/post-intervention [41,47,48]. Future interventions should therefore focus on the optimal period for initiation or the duration and intensity of the intervention in order to assess the biggest impact on weight reduction and consider meaningful retention. They should also include weight loss maintenance follow-up beyond 12 months, which is the key to longer-term clinical outcomes for the mother [61].

Interventions were delivered by individuals with varying qualifications. This contrasts with a recent review which summarised successful interventions focusing on lower gestational weight gain in overweight and obese women, where the majority of interventions were delivered by primary care providers (e.g., GPs and midwives) [62]. In the postpartum period, routine appointments with healthcare professionals are less frequent, than in the antenatal period. Future studies should explore the mechanism of delivery of the intervention and consider who is best placed to deliver interventions during the postpartum period.

Interestingly, only one effective intervention reported the use of a lifestyle app [45], other successful trials used an online platform, MyPyramid, which provided individualised advice on weight loss and dietary management [42]. or a web-based lifestyle modification programme [49]. In four studies, participants received individualised dietary prescriptions, personalised feedback via inter-visit telephone calls [41,47,52] and individualised positive-reinforcement messages through cell-phone texts [39]. New technology presents a novel method to tailor lifestyle advice during the postpartum period. Previous research has shown that individualising an intervention was more likely to be associated with efficacy [63,64]. Future trials should consider the use of app-based technology as an additional technique to aid weight loss, particularly in light of a previous systematic review which highlighted that app-based interventions have the potential to support women throughout their pregnancy [65]. Together, this suggests that a flexible approach to interventions with sustained contact is advisable in the postpartum period as it may facilitate wider engagement and adherence to the intervention; similar findings have recently been reported in a literature review on weight loss after pregnancy [58].

One limitation of this systematic review was the level of missing information in the published reports due to the drop-out rates from randomisation to the study end points. Attrition is a particular issue for postpartum women [66] and in randomised controlled trials in general [67]. In this review, only one of the trials reporting on an effective intervention described a high dropout rate [43]. In this case, it was attributed to a proportion of women becoming pregnant again, and to women in the control arm being dissatisfied with their intervention allocation [43]. For the nine unsuccessful trials reported here, the attrition rate was high in seven; therefore, the non-significant results of these trials may have been due to the high dropout rate. Disparities were notable across the studies, particularly regarding participant characteristics and also the type, setting, intensity and duration of the intervention. Furthermore, a small number of studies reported on the association between breastfeeding and PPWR. Future trials should consider reporting this association, as it may be a successful strategy to promote weight management in overweight and obese postpartum women. This review was limited by the heterogeneity between the study designs and intervention types, precluding meta-analysis. Further limitations include the small sample sizes of the included studies, which can affect power and reliability of the data [68]. Another limitation of this review was that only studies published in English were analysed.

## 5. Conclusions

Effective strategies to support the management of postpartum weight are warranted to support weight regulation in overweight and obese women of reproductive age and beyond. Evidence from this review suggests that in order to reduce postpartum weight, short, intensive interventions including diet or diet and physical activity, commencing in the postpartum period, may be effective. Further research is needed to understand methods of long-term weight maintenance, intervention type and duration. There was insufficient evidence in this review to conclude the effectiveness of interventions targeting postpartum weight retention which were initiated during the antenatal period. Future intervention trials, with comparable study designs and trials comparing pregnancy-only to postpartum-only interventions, are required to determine the ideal approach to support healthy weight attainment in overweight and obese women in the postpartum period.

## Figures and Tables

**Table 1:** Summary of population, intervention, comparison, outcomes and study design (PICOS) criteria for the inclusion of interventions.

Selection Criteria	Inclusion Criteria
Participants	Pregnant or postpartum (up to 2 years after birth) women, with a BMI ≥ 25 kg/m^2^ and ≥18 years old
Intervention	Diet or diet and physical activity initiated during pregnancy, postpartum or both
Comparison	Standard care
Outcomes	Maternal postpartum weight or body composition data >3 months and <2 years after delivery
Studies	Randomised controlled trials

**Table 2:** Characteristics of the antenatal/postnatal interventions.

Author/Year Trial Name Country	Aims	Timing of Intervention	Reported Primary/Secondary Outcomes	Study Population	Intervention
**Pregnancy only**					
**Phelan et al. 2013** **[35]** **Fit for Delivery** **USA**	To determine if a behavioural intervention initiated during pregnancy could decrease the proportion of women who exceeded the recommendations for GWG and increase the proportion of women who returned to pre-pregnancy weight by 6-month PP	Commenced 10–16 weeks gestation Ended at birth	Proportion of women at or below pre-pregnancy weight at 6 months PP Secondary outcome: Proportion of women who exceeded 1990 IOM recommended GWG	*N* = 401 all BMI I: *n* = 201 26.32 ± 5.6 kg/m^2^ C: *n* = 200 26.48 ± 5.9 kg/m^2^ Subgroup analysis was provided for women with BMI ≥25.0 kg/m^2^: *n* = 200	I: Diet and physical activity - Standard care plus a behavioural lifestyle intervention which involved one face-to-face visit with an interventionist; weekly mailed materials that promoted appropriate weight gain, healthy eating (20 kcal/kg) and exercise (30 min of walking most days/week); individual graphs of weight gain; and telephone-based feedback C: Usual care included nutrition counselling in line with the WIC program
**Vesco et al. 2016** **[36]** **Healthy Moms Trial** **USA**	To determine if women who received a weight management intervention during pregnancy weighed less at 1-year PP	Commenced <20 weeks gestation Ended at birth	Weight change at 1-year PP, defined as weight at 12-months PP minus weight at randomisation	*N* = 114 OB women only I: *n* = 56 BMI 36.7 ± 5.2 kg/m^2^ C: *n* = 58 BMI 36.8 ± 5.2 kg/m^2^	I: Diet and physical activity - Two individual dietary counseling sessions with an interventionist - Weekly group meetings - Daily food and activity diaries (reviewed weekly) - Target: Maintain weight within 3% of randomisation weight by 12 months pp - Keep calorie intake within an individual goal and adopt a sodium-restricted diet - Exercise daily (goal of 30 min moderate activity per day or 10,000 steps per day on pedometer) C: Usual care included a single dietary advice session with a dietitian.
**Vinter et al. 2014** **[37]** **LiP study** **Denmark**	To determine the effect of a lifestyle intervention in pregnancy on PPWR at 6 months and the association between breastfeeding and PPWR	Commenced <14 weeks gestation Ended at birth	PPWR, defined as 6-month PP weight minus weight at study inclusion	304 OB women only I: *n* = 150 33.4 kg/m^2^ (31.7–36.5) C: *n* = 154 33.3 kg/m^2^ (31.7–36.9)	I: Diet and physical activity - Four individual dietary counselling sessions with a dietitian - Counselling on postpartum diet, nutritional requirements during breastfeeding and weight loss - 1 h/week aerobic classes - Free fitness membership C: Usual care
**Pregnancy and postpartum**					
**Ferrara et al. 2011** **[38]** **DEBI study** **USA**	To evaluate the feasibility of a prenatal/postpartum intervention to modify diet and physical activity	Commenced at diagnosis of GDM Ended 12 months PP	Proportion of women who reached their weight goal by 12 months PP, (reduction to 5% below their pre-pregnancy weight)	*N* = 197 all BMI Subgroup analysis was provided for women with BMI ≥25.0 kg/m^2^ *n* = 114 I: *n* = 96 All BMI OW = 24% (*n* = 23) OB = 35% (*n* = 34) C: *n* = 101 OW = 22% (*n* = 22) OB = 35% (*n* = 35)	I: Diet and physical activity 3 intervention phases delivered by dietitians Prenatal: GDM diagnosis to 6 weeks PP - 1 in-person session, 2 telephone calls - Encouraged to follow the ADA diet - Engage in moderate intensity physical activity for 150 min/week. - Advice on breastfeeding for 6 months Postpartum: 6 weeks to 7 months PP - Goal of a 5% reduction in pre-pregnancy weight by 12 months PP - 2 individualised in-person sessions and telephone calls - 150 min of moderate or vigorous physical activity/week. - Consume 25% or less of total calories from fat per day. Maintenance phase: 7 to 12 months PP - 3 telephone calls to reinforce the behavioral changes and address relapses. C: Usual care received materials on GDM, infant safety and general health
**Herring et al. 2017** **[39]** **USA**	To determine whether an early pregnancy behavioral intervention could increase the proportion of obese African American women who were at or below their early pregnancy weights by 6- and 12-months PP	Commenced <20 weeks gestation Ended 6 months PP	Number of women at or below their early pregnancy weights by 6- and 12-months PP	*N* = 66 OW/OB African American women I: *n* = 33 BMI 33.5 ± 5.8 kg/m^2^ C: *n* = 33 BMI 32.2 ± 5.4 kg/m^2^	I: Diet and physical activity Behavioural lifestyle intervention designed to: (1) Prevent excessive GWG (delivered from baseline to 36 weeks’ gestation) (2) Promote weight loss postpartum (delivered between 10 weeks and 6 months PP) Behaviour change goals included ‘limit junk and high fat foods to no more than 1 per day’, ‘walk 5000 steps daily’ and ‘weigh yourself weekly’. Delivered via three mechanisms: - Daily text messages tailored to a behavioural goal - Weekly Facebook posts with links to websites and videos - Weekly to monthly scripted calls with a health coach - Participants were provided with digital scales, pedometers, water bottles and portion plates. C: Usual care
**Peccei et al. 2017** **[40]** **USA**	To assess the effect of a culturally-appropriate nutritional intervention on GWG and PPWR	Commenced <16 weeks gestation Ended 6 months PP	Weight retention at 6 months PP from baseline	*N* = 300 OW/OB women I: *n* = 200 *n* = 85 OW *n* = 115 OB White: 36%, Black: 8%, Hispanic: 49%, Other: 7% C: *n* = 100 *n* = 43 OW *n* = 57 OB White: 46%, Black: 3%, Hispanic: 43%, Other: 8%	I: Diet only - Pregnancy: culturally appropriate, 10–30 min bi-monthly counselling on a low carbohydrate diet with a dietitian either in person or by phone - Pregnancy-specific individualised meal plans, weight gain trajectory assessments, counselling on food labels, shopping for healthy foods, calorie comparisons and supplement advice - Postpartum (6 weeks to 6 months): nutritional assessment and individualised meal plans for postpartum period; adjusted according to breastfeeding C: Standard prenatal and postnatal care
**Postpartum only**					
**Bertz et al. 2012** **[41]** **Sweden**	To evaluate whether a 12-week dietary, physical exercise or combined dietary and physical exercise behaviour modification reduces body weight in lactating women	Commenced 10–14 weeks PP Duration 12 weeks	To measure changes (by DXA scan) from baseline in body weight and body composition	*N* = 68 OW/OB women (calculated from pre-pregnancy weight) I: *n* = 51 Diet only: *n* = 17 BMI 30.0 ± 2.6 kg/m^2^ Exercise only: *n* = 18 BMI 30.4 ± 3.1 kg/m^2^ Diet and exercise: *n* = 16 BMI 29.9 ± 2.2 kg/m^2^ C: *n* = 17 BMI 30.2 ± 3.4 kg/m^2^ BMI data from baseline	I: Diet and physical activity Individual behaviour modification face to face counseling totaling 5 h. D: Reduction of 500 kcal/day - Limit sweets and snacks to 100 g/week - Substitute low-fat and low-sugar alternatives for regular foods - Cover one-half of the plate with vegetables at lunch - Reduce portion sizes E: 45-min brisk walk 4 day/week at 60–70% of the maximum heart rate. Women were contacted biweekly with text messages. C: Usual care
**Colleran et al. 2012** **[42]** **USA**	An intervention examining the effects of energy restriction and exercise on body composition in overweight/obese lactating women	Commenced < 4 weeks PP Ended 20 weeks after recruitment	Primary outcome: To improve total diet or overall pattern of food consumptionAdditional outcomes: Changes in anthropometric and body composition, including BMI	*N* = 31 OW/OB (calculated at baseline) Data available for *n* = 27 I: *n* = 14 BMI 29.7 ± 3.6 kg/m^2^ C: *n* = 13 BMI: 28.0 ± 3.3 kg/m^2^	I: Diet and physical activity - A 16-week intervention using MyPyramid to estimate energy needs based on age, weight, height, and lactation status. - Printout generated which graphically displayed their recommended energy needs and food group servings Research assistants travelled to participants’ homes up to three times/week to facilitate the exercise session and dietary counselling C: Usual care group were asked not to participate in structured exercise or decrease their energy intake during the 16-week intervention
**Craigie et al. 2011** **[43]** **WeighWell** **UK**	To evaluate the feasibility of a weight loss intervention in socially disadvantaged women	Commenced 6–18 months PP Duration 12 weeks	To assess changes in body composition (weight, WC and skinfolds), dietary intake and physical activity	Socially disadvantaged women. *N* = 52 OW/OB calculated at baseline I: *n* = 29 BMI 31.6 ± 4.7 kg/m^2^ C: *n* = 23 BMI 31.6 ± 5.4 kg/m^2^	I: Diet and physical activity - 3 × monthly face-to-face consultations with a trained lifestyle counselor - 3 structured telephone calls - 500 kcal/day reduction - 150 min of moderate-vigorous activity per week C: Written information on weight loss and usual care
**Falciglia et al. 2017** **[44]** **USA**	To evaluate the effectiveness of a dietary intervention to increase target vegetable intake in OW PP women	Commenced 6 weeks PP Duration 12 months	Primary outcome: Maternal dietary intake Additional outcomes: Changes in body composition, including BMI	*N* = 104 OW/OB calculated at baseline I: *n* = 52 C: *n* = 52	I: Diet only - Mothers received 4 × 60 min education sessions with a nutrition professional and 8-month follow-up phone calls. - Sessions were tailored to SES of the participant and included advice on vegetable intake for the mother and her infant - Dietary guidelines were based on MyPyramid C: Usual care with standard educational material on MyPyramid
**Gilmore et al. 2017** **[45]**	E-Moms: A personalized mHealth intervention for health and weight loss in postpartum women	Commenced <8 weeks PP Duration 16 weeks	Primary outcomes: Change in weight Additional outcome: Changes in body fat percentage, waist circumference, hip circumference and waist-to-hip ratio	*N* = 40 OW/OB Calculated at baseline Data available for *n* = 35 I: *n* = 19 BMI 31.3 ± 3.2 kg/m^2^ C: *n* = 16 BMI 32.7 ± 2.8 kg/m^2^	I: Diet and physical activity - The participants were given access to the SmartLoss phone application and an iPhone - The application included real-time weight and activity monitoring, scheduled delivery of health information, and interventionist feedback - To track weight and activity, participants were provided a BodyTrace scale and a Fitbit Zip accelerometer. Weight and steps were plotted on a weight and step graph found in the SmartLoss App and interventionist web portal once daily Target goals included weight loss of 10 ± 3% of enrolment weight at 16 weeks. An initial step goal was 500 steps/day which increased by 500 steps per day each week. 16 SmartTips were automatically sent each week and included tips on diet, physical activity and behaviour modification C: Standardised advice and services for postpartum nutrition and weight management through their WIC clinic
**Herring et al. 2014** **[46]** **Healthy4Baby** **USA**	To examine the feasibility, acceptability, and efficacy of a technology-based weight loss intervention for urban, low-income mothers	Commenced 2 weeks–12 months PP Duration 14 weeks	Change in body weight from baseline to the end of the intervention	*N* = 18 OW/OB calculated from pre-pregnancy weight I: *n* = 9 BMI 36.9 ± 6.1 kg/m^2^ C: *n* = 9 BMI 36.9 ± 6.1 kg/m^2^	I: Diet and physical activity Daily text messages and biweekly calls on energy deficit, setting personal goals around behaviour change strategies - Limit sugary drinks to no more than 1 per day - Limit junk and high fat foods to no more than 1 per day - Aim for 1200–1500 kcal/day, - Walk 30 min or 5000 steps every day - Self-monitoring texts 3/4 × week to probe about adherence to behavioral strategies - Calls conducted biweekly C: Usual care
**Huseinovic et al. 2016** **[47]** **LEVA in Real Life Study** **Sweden**	To evaluate short and long-term effectiveness of diet behaviour modification for weight loss in women	Commenced 6–15 weeks PP Duration 12 weeks	Change in body weight from baseline. Additional outcomes include BMI, WC, HC, body fat %, dietary intake and physical activity at the end of the intervention and 1-year PP	*N* = 110 OW/OB Baseline BMI ≥ 27 kg/m^2^ I: *n* = 54 BMI 31.8 ± 4.0 kg/m^2^ C: *n* = 56 BMI 31.6 ± 3.4 kg/m^2^	I: Diet only - 1.5 h face-to-face visit with the dietitian - Reduction of 500 kcal/day - Limit sweets, salty snacks, and caloric drinks to 1 day/week and a max of 100 g/week - Substitute regular foods with low-fat and/or low-sugar alternatives - Cover one-half of the plate with vegetables - Reduce portion sizes - Phone calls and text messages were used throughout the trial to keep in contact - Weight loss goal of 0.5 kg/week; final loss of 6 kg after 12 weeks C: Usual care and a brochure on healthy eating
**Lovelady et al. 2000** **[48]** **USA**	To determine whether weight loss by women during lactation affects the growth of their infants	Commenced 4 weeks PP Duration 10 weeks	Primary outcome: growth of infant. Additional outcomes include: maternal weight, BMI, body fat % and skinfolds	*N* = 40 OW only Baseline BMI 25–30 kg/m^2^ I: *n* = 27 27.6 ± 2.4 kg/m^2^ C: *n* = 21 28.0 ± 2.1 kg/m^2^	I: Diet and physical activity - Energy intake reduction of 500 kcal/d - Aerobic exercise (4×/week of 45-min sessions at 65–80% maximal heart-rate) - Weight loss goal: 0.5 to 1.0 kg/ week C: Instructed to: - Not restrict energy intake - Not perform vigorous aerobic exercise more than once per week All women were given a multivitamin supplement to take which contained at least 50 percent of the recommended dietary allowances for lactating women
**Nicklas et al. 2014** **[49]** **Balance after Baby** **USA**	To test the feasibility and effectiveness of a web-based lifestyle intervention for women with recent GDM to reduce weight retention	Commenced 6 weeks PP Duration 24 weeks	Change in body weight at 12 months from (a) baseline visit and (b) self-reported pre-pregnancy weight	*N* = 75 OW/OB I: *n* = 36 31.2 ± 5.8 kg/m^2^ C: *n* = 39 31.6 ± 5.5 kg/m^2^	I: Diet and physical activity Web-based lifestyle modification program, including advice on how to achieve the following: - Lower glycemic index - Higher fibre - Controlled portion sizes - Gradually increasing physical activity to ≥150 min/week - Weight goal: return to pre-pregnancy weight C: Usual care plus distribution of a handout
**Østbye et al. 2009** **[50]** **Active mothers postpartum** **USA**	To promote a reduction in BMI up to 24-months PP via sustainable lifestyle changes	Commenced 6 weeks PP Duration 9 month	Changes from baseline to 1-month post-intervention in: diet, physical activity and weight	*N* = 450 OW/OB at baseline I: *n* = 225 BMI 33.1 ± 6.7 kg/m^2^ C: *n* = 225 BMI 32.9 ± 6.0 kg/m^2^	I: Diet and physical activity - 8 healthy-eating classes - 10 physical-activity classes Delivered by a health counsellor 6 telephone-counseling sessions covering: - Reducing total caloric intake (decrease in calorie-dense foods) - An increase in fruit and vegetable consumption - Increasing physical activity to 30 min/day × 5/week 6-months postpartum, a sport stroller was provided to encourage walking for exercise outside of class and after the end of the intervention. C: Received biweekly newsletters with general tips for postpartum mothers
**Wilkinson et al. 2015** **[51]** **TRiM** **Australia**	To evaluate a PP weight management programme on weight loss	Commenced 6-weeks PP Ended 6 months PP	Weight loss from pre-pregnancy to 6 months PP and from 6 weeks PP to 6 months PP	*N* = 81 OW/OB I: *n* = 40 BMI 33.5 ± 5.9 kg/m^2^ C: *n* = 41 BMI 33.5±x6.4 kg/m^2^	I: Diet only 6-month intervention 36 weeks gestation: 1-h face-to-face nutrition assessment with a dietitian, goal-setting introduction and counselling session regarding nutrition post-pregnancy 6 weeks until 3 months postpartum: Every 2 weeks information and goal-setting sheets posted 3 months until 6 months postpartum: monthly information and goal-setting sheets posted C: Usual care, at 36 weeks gestation received nutrition for breastfeeding resource
**Wiltheiss et al. 2013** **[52]** **KAN-DO** **USA**	To improve diet and physical activity habits of mothers, to promote PP weight loss	Commenced 2–7 months PP Duration 10 months	Primary outcomes: Changes in diet quality and reduced energy intake from baseline Secondary outcomes: weight loss from baseline	*n* = 392 OW/OB calculated from pre-pregnancy BMI Available for analysis: I: *n* = 131 C: *n* = 145 weight: 87.5 kg ± 15.7	I: Diet only - 8 monthly educational kits via mail - Kits focused specifically on changing dietary habits, appropriate portion size, ways to increase fruit and vegetable intake, ideas for nutritious snacks, how to read food labels and sample grocery lists with meal plans - Participants received a 20–30 min phone call with a health coach to discuss the content and address motivations and barriers C: Participants in the control arm received monthly newsletters focusing on reading skills and enjoyment of the preschooler.

Abbreviations: %: percentage; ADA: American Diabetes Association; BMI: body mass index; C: control; DXA; dual energy X-ray absorptiometry; E: exercise; GDM: gestational diabetes mellitus; HC; hip circumference; GWG: gestational weight gain; I: intervention; IOM; Institute of Medicine; kcal: kilocalories; *n*: number; kg: kilograms; OB: obese; OW: overweight; PP: postpartum; PPWR: postpartum weight retention; SES: socioeconomic status; WC: waist circumference; WIC: Women, Infant and Children; wk: week.

**Table 3:** Impact of interventions on maternal body composition.

Reference	Postpartum Weight Outcome	Additional Outcomes	Follow-Up
**Pregnancy only**			
Phelan et al. 2013 [35]	The percentage of NW and OW/OB women who returned to their pre-pregnancy weight or below by 6 months PP was significantly greater in the I vs. C group (OW/OB = I: 25.6%, C: 16.7%, *p* = 0.005) Net weight retention at 6 months PP for all women I and C: 3.7 ± 5.9 kg vs. 4.3 ± 6.2 kg	The intervention had no effect on GWG for OW/OB women The I and C groups had similar rates of breastfeeding at 6 months. Breastfeeding was related to higher odds of achieving 6-month preconception weights or below (OR: 2.4; 95% CI: 1.4, 4.2; *p* = 0.002)	*N* = 177 OW/OB 6-months PP
Vesco et al. 2016 [36]	There was no significant difference between I and C in change in weight from randomisation to 1-year PP, mean change: −0.5 (−4.0 to 3.1). 56% and 58% of the I and C were at or below their pre-pregnancy weight	GWG was positively associated with PPWR (*b* = 0.8 kg, 95% CI: 0.4 to 1.3, *p* < 0.001)	*N* = 89 1-year PP
Vinter et al. 2014 [37]	There was no significant difference in PPWR between I and C	There was significantly lower PPWR in women with GWG <9 kg in line with the IOM recommendations (*p* = 0.001) Breastfeeding significantly negatively influenced PPWR (regression coefficient −2.64, *p* = 0.002)	*N* = 238 I: 46%, C: 58% 6 months PP
**Pregnancy and Postpartum**			
Ferrara et al. 2011 [38]	Percentage of women reaching PP weight goal was not significantly different between the I and C groups at 7- and 12-months PP (32.5% vs. 22.0%, *p* = 0.41) and (30.0% vs. 14.6%, *p* = 0.15)	At 12 months PP women in the intervention with GWG within IOM recommendations were more likely to reach weight goals (*p* = 0.04) compared to women who exceeded the IOM guidelines during their pregnancy. Intervention participants showed higher, but not significant, rate of partial or exclusive breastfeeding (*p* = 0.09)	*N* = 110 12 months PP
Herring et al. 2017 [39]	At 6 months PP the I group were significantly more likely to be at or below their pre-pregnancy weight (56% vs. 29%, *p* = 0.04), there was no difference at 12 months PP (*p* = 0.83)	GWG was positively associated with postpartum weight change at 6 months, (*p* = 0.006). This association was not maintained by 12 months PP (*p* = 0.30)	*N* = 56 I: *n* = 29, C: *n* = 27 12 months PP
Peccei et al. 2017 [40]	At 6 months PP, there was no significant difference between the I and C groups for PPWR	OW women in the intervention group had a significantly lower percent of initial BMI at 6 months PP (*p* = 0.026) (101% compared with 106%)	*N* = 104 I: *n* = 63, C: *n* = 41 6 months PP
**Postpartum only**			
Bertz et al. 2012 [41]	Treatment group D, and not treatment group E, caused a significant reduction in weight and fat mass at the end of the intervention (both *p* < 0.001) and 1-year follow-up (*p* < 0.001 and *p* = 0.002, respectively)	No significant associations between treatment groups and breastfeeding	I: D: *n* = 15 E: *n* = 16 DE: *n* = 16 C: *n* = 15 12 months PP
Colleran et al. 2012 [42]	Significant differences in energy intake (−613 kcal vs. −171 kcal; *p* = 0.03), saturated fat intake (*p* < 0.01), and % of energy from added sugars (*p* < 0.01) were found between I and C groups	The Intervention group lost significantly more weight between baseline and the end of the intervention (−5.8 [3.5] kg) than the C group (−1.6 [5.4] kg) (*p* = 0.03) All women were breast feeding throughout the trial	I: *n* = 14 C: *n* = 13 20 weeks PP
Craigie et al. 2011 [43]	The I group had a significantly lower BMI and body fat percentage at follow-up compared to baseline (*p* = 0.009 and *p* = 0.029, respectively)	No relevant additional outcomes reported	I: *n* = 22 C: *n* = 14 2 years PP
Falciglia et al. 2017 [44]	From baseline there was no difference in BMI for I and C at the end of the intervention and subsequent follow-up	Mothers in the I group had a higher reported consumption of vegetable intake at 6, 12- and 18-months PP Similar breastfeeding rates between the groups (*p* = 0.42)	I: *n* = 32 C: *n* = 35 12- and 18-months PP
Gilmore et al. 2017 [45]	From week 0 to week 16 there was no difference in body weight change, WIC Moms vs. E-Moms; +1.8 ± 0.9 vs. −0.1 ± 0.9 kg; *p* = 0.10.	Weight change did not significantly differ between those who breastfed during the study	I: *n* = 19 C: *n* = 16 4 months from baseline
Herring et al. 2014 [46]	Intervention participants had significantly greater weight loss between baseline and the end of the trial (−3.2 kg, 95% CI: −6.2, −0.1 kg, *p* = 0.04)	Greater reduction was observed in sugary drink, fast food and chip consumption in the intervention arm 22% of the cohort breastfed	I: *n* = 9 C: *n* = 9 12 months PP
Huseinovic et al. 2016 [47]	At the end of the intervention the I group weight was 6.1 kg lower (−8.4, −3.2 kg) compared with 1.6 kg lower (−3.5, −0.4 kg) in the C group (*p* < 0.001)	The difference was maintained at the 1-year follow-up for the I group, −10.0 kg (−11.7, −5.9 kg) compared with −4.3 kg (−10.2, −1.0 kg) in the C group (*p* = 0.004)	I: *n* = 47 C: *n* = 53 12 months PP
Lovelady et al. 2000 [48]	The DE group lost more weight (4.8 ± 1.7 kg vs. 0.8 ± 2.3 kg, *p* < 0.001) and fat mass (4.0 ± 2.0 kg vs. 0.3 ± 1.8 kg, *p* < 0.001) than the control group.	A greater percentage of women in the DE group were within 1 kg of their pre-pregnancy weight at 1-year follow up; All women were breast-feeding throughout the trial	I: *n* = 21 C: *n* = 19 12 months PP
Nicklas et al. 2014 [49]	The I group lost a mean of 2.8 kg from 6 weeks to 12 months PP. Women in the control group gained an average of 0.5 kgs (−1.4 to +2.4 kg). This was a statistically significant difference (*p* = 0.022)	Women in the intervention were closer to pre-pregnancy weight at 12 months PP than women in the C group. (*p* = 0.035) There were no differences in breastfeeding rates	I: *n* = 36 C: *n* = 39 12 months from baseline
Østbye et al. 2009 [50]	From 6 weeks PP to 1-month post intervention there was no significant difference between mean weight loss in the I group and C group	Class participation was significantly associated with weight change (*p* = 0.01), but not with change in diet or physical activity	I: *n* = 171 C: *n* = 160 12 months PP
Wilkinson et al. 2015 [51]	No significant differences were observed between any outcomes	There was a low amount of data collected at various time points; therefore, the study was underpowered to detect any difference between the study arms. Women in the I arm breastfed for half a month longer (180 vs. 164 days, *p* = 0.10)	I: *n* = 24 C: *n* = 17 6 months PP
Wiltheiss et al. 2013 [52]	From 5 months PP to 10 months follow-up, the intervention did not result in significant weight loss	Diet quality was significantly related to weight change from 5 to 15 months postpartum (*p* < 0.01) No significant relationship was found between lactation score and weight change	I: *n* = 131 C: *n* = 145 10 months from baseline

Abbreviations: BMI: body mass index; C: control; D: diet only; DE: diet and exercise; E: exercise only; GWG: gestational weight gain; I: intervention; IOM; Institute of Medicine; kcal: calories; *n*: number; kg: kilograms; NW: normal weight; OB: obese; OR: odds ratio; OW: overweight; PP: postpartum; PPWR; postpartum weight retention.

**Table 4:** Summary of incomplete data and sources of bias for all included studies.

Author	Randomization	Allocation	Attrition	Overall Risk of Bias
Phelan et al. 2013 [35]	LOW	LOW	LOW	LOW
Vesco et al. 2016 [36]	LOW	UNCLEAR	HIGH	MODERATE
Vinter et al. 2014 [37]	LOW	LOW	HIGH	MODERATE
Ferrara et al. 2011 [38]	LOW	LOW	LOW	LOW
Herring et al. 2017 [39]	LOW	LOW	LOW	LOW
Peccei et al. 2017 [40]	LOW	LOW	HIGH	MODERATE
Bertz et al. 2012 [41]	LOW	LOW	LOW	LOW
Colleran et al. 2012 [42]	LOW	UNCLEAR	LOW	LOW
Craigie et al. 2011 [43]	LOW	LOW	HIGH	MODERATE
Falciglia et al. 2017 [44]	UNCLEAR	UNCLEAR	HIGH	HIGH
Gilmore et al. 2017 [45]	UNCLEAR	UNCLEAR	LOW	MODERATE
Herring et al. 2014 [46]	LOW	LOW	LOW	LOW
Huseinovic et al. 2016 [47]	LOW	LOW	LOW	LOW
Lovelady et al. 2000 [48]	LOW	UNCLEAR	LOW	LOW
Nicklas et al. 2014 [49]	LOW	LOW	LOW	LOW
Østbye et al. 2009 [50]	LOW	UNCLEAR	HIGH	MODERATE
Wilkinson et al. 2015 [51]	LOW	LOW	HIGH	MODERATE
Wiltheiss et al. 2013 [52]	UNCLEAR	UNCLEAR	HIGH	HIGH

## Appendix A

**Table A1:** MEDLINE (1946–23 January 2018) and EMBASE (1946–23 January 2018). (P) Patient.

#1	pregnancy/
#2	pregancy.mp.
#3	pregnan*.tw.
#4	gestation/
#5	maternal.mp.
#6	exp postpartum/
#7	exp puerperium/
#8	postnatal care.mp
#9	weight gain/
#10	exp body composition/
#11	exp body mass index/
#12	body size/
(I) Intervention	
#13	exp animals/not humans.sh.
#14	exp Diet/
#15	intervention studies/
#16	education.mp.
#17	counceling/or counseling.mp.
#18	Energy Intake/or dietary intake.mp.
#19	nutrition* advice/or diet* advice.mp.
#20	low energy/or low calorie.mp.
#21	glyc?emic index/or glyc?emic load.mp.
#22	low carbohydrate.mp.
#23	low fat.mp.
#24	dietitian/or dietician/or nutritionist.mp.
#25	dietary assessment/or dietary report.mp.
#26	diet* recall.mp.
#27	food frequency questionnaire.mp.
#28	food diary/or food record/or diet record.mp.
#29	health* eating.mp.
(O) Outcome	
#30	exp Weight Gain/
#31	body weight management.mp.
#32	weight reduction.mp.
#33	weight change.mp
#34	skinfold.mp.
#35	body mass index.mp.
#36	body composition.mp.
#37	exercise.mp.
#38	#1 OR #2 OR #3 OR #4 OR #5
#39	#6 OR #7 OR #8 OR #9 OR #10 OR #11 OR #12
#40	#14 OR #15 OR #16 OR #17 OR #18 OR #19 OR #20 OR #21 OR #22 OR #23 OR #24 OR #25 OR #26 OR #27 OR #28 OR #29
#41	#30 OR #31 OR #32 OR #33 OR #34 OR #35 OR #36 OR #37
#42	#38 AND #39 AND #40 AND #41
#43	#42 NOT #13
#44	#43 and 2000:current
Total: 2456	

**Table A2:** Cochrane Central Register of Controlled Trials (CENTRAL) in the Cochrane Library.

#1	pregnan*
#2	postpartum
#3	gestation
#4	Weight gain
#5	body mass index
#6	diet
#7	lifestyle
#8	Physical activity
#9	intervention
#10	Follow up
#11	Weight retention
#12	skinfold
#13	animal not human
#14	#1 OR #2 OR #3
#15	#4 OR #5
#16	#6 OR #7 OR #8 OR #9
#17	#10 OR #11 OR #12
#18	#14 AND #15 AND #16 AND #17
#19	#18 NOT #13
Articles cited: 277	
